# Assessment of the SABAS cutoff point based on studies in problematic smartphone use

**DOI:** 10.1192/j.eurpsy.2025.2190

**Published:** 2025-08-26

**Authors:** C. Sándor, N. Pirwani, M. Csibi, A. Szabo

**Affiliations:** 1 Faculty of Psychology, Department of Sciences and Letters, George Emil Palade University of Medicine, Pharmacy, Science, and Technology, Targu Mures, Romania; 2 Institute of Health Promotion and Sport Sciences, Faculty of Education and Psychology, ELTE Eötvös Loránd University, Budapest; 3 Institute of Special Education, Faculty of Pedagogy, Eszterházy Károly Catholic University, Eger, Hungary

## Abstract

**Introduction:**

The SABAS is a single-factor measure of problematic smartphone use, with higher scores indicating a potential addictive tendency. Some researchers (Peng et al., 2023) suggest a cutoff point of 23 out of a maximum available score of 36. Other researchers consider a high mean a guideline without suggesting a possible threshold. This score will indicate the presence of the problematic factor under investigation, regardless of age.

**Objectives:**

Our study aims to identify a score for problematic smartphone use that may already indicate vulnerability to addiction. The research investigates the proposal of a possible cutoff point for problematic smartphone use based on several SABAS surveys over 9 years.

**Methods:**

In our research, 1228 participants completed four online surveys between 2015 and 2023. The age distribution was 9-73 years, with 41.2% male and 58.8% female. Our research instrument was the Smartphone Application-Based Addiction Scale (Csibi et al., 2018) questionnaire, a 6-question questionnaire designed to detect problematic smartphone use. We hypothesized that SABAS scores that show a significant relationship with cutoff scores of clinical questionnaires with convergent validity (NMP-Q, SPAI, SHAI, FNPQ) could be as cutoff scores within the measure.

**Results:**

Our results showed a significant correlation between SABAS and NMP-Q scores (*r*(238) = .63, *p* = .001), with the mean of the moderate-severity nomophobia score (88.5 points) being the mean of the SABAS 23 score. For the response distribution corresponding to the NMP-Q prevalence of severe nomophobia (100 points or more), the SABAS score mean was 29 points. The mean scores on the SPAI questionnaire were 85.82 (*SD*=22.76) and 97.17 (*SD*=31.65), respectively, for the subscales Functional Impairment 22.47 (*SD*=8.41) and 27.29 (*SD*=9.59), Compulsive Behaviour 29.48 (*SD*=9,03) and 34.41 (*SD*=11.64), Withdrawal 21.77 (*SD*=6.41) and 23.41 (*SD*=8.55), and Tolerance 12.10 (*SD*=3.61) and 12.05 (*SD*=4.64). The correlation was also evident for the SHAI (*r*(439) = .67, *p* = .001), its subscales, and the FNPQ scale (*r*(398) = .27, *p* = .001).

**Conclusions:**

The mean SABAS score indicating problematic smartphone use was 23 points, with scores above this point indicating increasingly severe use of analyzed behavior. Those with a score of severe nomophobia scored 29 or higher on the SABAS scale. The SABAS shows a significant relationship with the cutoff scores of the convergent validity questionnaires along the mean scores indicated above (23,29), so we suggest using these scores as cutoffs.

**Disclosure of Interest:**

None Declared

